# Augmentation strategies for treatment-refractory clozapine patients

**DOI:** 10.1192/j.eurpsy.2021.109

**Published:** 2021-08-13

**Authors:** O. Freudenreich

**Affiliations:** Mgh Schizophrenia Clinical And Research Program, Massachusetts General Hospital, Boston, United States of America

**Keywords:** treatment-resistant schizophrenia, Augmentation strategies, clozapine

## Abstract

**Background:**

Clozapine can be a life-saving and course-altering treatment for patients with psychosis, particularly treatment-resistant schizophrenia. Unfortunately, clozapine monotherapy rarely leads to a full symptomatic remission.

**Aims:**

This talk outlines key decision points in the use of clozapine: how to select patients for clozapine treatment and how to optimize clozapine’s efficacy in patients with a poor response to an adequate clozapine monotherapy trial.

**Conclusions:**

Clozapine’s main indication is for treatment-resistant schizophrenia. Therapeutic drug monitoring (TDM) should be used to optimize clozapine dosing during a clozapine trial and to rule-out pseudo-resistance. Up to 50% of patients do not respond to clozapine monotherapy and augmentation strategies can be utilized in such cases. Pharmacological add-on treatments are selected based on the most prominent symptom cluster (refractory psychosis, negative symptoms, depression and suicidality, aggression). Electroconvulsive therapy is the most effective augmentation strategy for refractory psychosis and suicidality. Non-pharmacological interventions and a focus on quality of life become important considerations in clozapine non-responders.

**Comments:**

Clozapine is an important and underutilized tool in the management of treatment-resistant schizophrenia. It should be offered timely, as soon as treatment-resistance becomes apparent. Clinicians can use personalized augmentation strategies as part of a comprehensive treatment plan in order to achieve improvements even in patients with a poor response to clozapine alone. However, polypharmacy should be used judiciously, keeping in mind medical morbidity and quality of life.

**Disclosure:**

I have the following financial relationship with a commercial interest to disclose (recipient SELF; content area SCHIZOPHRENIA): Alkermes – Research grant (to institution), consultant honoraria (Advisory Board); Avanir – Research grant (to institution);

